# Urban children’s connections to nature and environmental behaviors differ with age and gender

**DOI:** 10.1371/journal.pone.0255421

**Published:** 2021-07-29

**Authors:** Ryan J. Keith, Lisa M. Given, John M. Martin, Dieter F. Hochuli

**Affiliations:** 1 School of Life and Environmental Sciences, The University of Sydney, Sydney, NSW, Australia; 2 Centre for Design Innovation, Swinburne University of Technology, Hawthorn, VIC, Australia; 3 Taronga Institute of Science & Learning, Taronga Conservation Society Australia, Mosman, NSW, Australia; Forest Research, UNITED KINGDOM

## Abstract

Global conservation is increasingly reliant on young people forming meaningful connections with urban nature. However, interactions with nearby nature do not inspire all children and adolescents living in cities to act pro-environmentally. Our survey of over 1,000 school students from Sydney, Australia, revealed that 28% of respondents maintained strong nature connections. Younger students (aged 8–11) were more strongly connected with nature than their older peers (aged 12–14), and environmental behaviors were negatively associated with increasing age. Differences between boys and girls were less consistent, resulting in part from differential functioning of questionnaire items. Regardless, girls were more willing than boys to volunteer for conservation. Our findings suggest that policies designed to strengthen urban children’s nature connections will be most effective if they explicitly address the “adolescent dip” and other emerging demographic patterns, thereby ensuring all young people reap the health, wellbeing, and conservation benefits of connecting with nature.

## Introduction

Humanity currently faces an unprecedented existential threat in the form of interconnected global crises: declining biodiversity and accelerating climate change [1]. These phenomena are caused by directly destructive human activities [2] and also arise as a result of our consumptive, reproductive, and democratic choices [3]. Thus, it is ultimately a crisis of human behavior that we are facing [4]. In response, researchers are seeking leverage points for changing people’s environmental behaviors [5].

Connectedness with nature is increasingly recognized as a potent motivator of people’s pro-environmental behaviors [6]. We connect with nature through sensory contact, emotion, beauty, meaning, and compassion [7], and the strength of an individual’s nature connection is a better predictor of environmental behavior than is their environmental knowledge [8]. This may be because behavioral commitment to conserving nature arises from affective and experiential nature connections in addition to cognitive ones [9]. The logic is clear: if people do not just know about, but also care about and personally know nature, then they will likely resolve to act in its benefit [9]. Crucially, the drivers of committed nature connection are interdependent, such that someone will most likely change their behaviors for the good of our planet if they experience a conscious, emotional, and embodied connection with nature rather than experiencing any one type in isolation.

Opportunities for establishing an experiential connection with nature may be declining. Currently, more than half of the global population lives in cities, with this habitation pattern changing the way we interact with nature [10]. The potential exists for an ‘extinction of [nature] experience’ to occur as cities continue to grow [11]. Urban densification can reduce the amount of accessible urban greenspace [12], causing a ‘nature deficit’ [13] in children who might otherwise be exposed to nature in such areas [14]. With limited opportunities to experience nature outside, urban youth can be restricted to interacting with indoor nature in the form of houseplants or domestic pets (where permitted); alternatively, they might only encounter the natural world vicariously through media or a window pane [15].

Children living in cities report fewer everyday interactions with nature than their rural counterparts [16]. This may be because few people interact consciously with urban nature, which is taken for granted, backgrounded, or rendered invisible by inattention blindness [17]. As a result, urban residents underestimate the hedonic benefits of time spent in nearby nature [18] and fail to perceive interactions with ‘mundane’ (vs. idealized, wild) nature as important or worthwhile [17]. This is a significant issue on both the day-to-day and lifetime scales, as nature experiences can confer health and wellbeing benefits [19]. Indeed, benefits extend beyond the individual, as the formation of an affinity for other species through interactions with urban nature can inspire conservation behaviors on a global scale [20]. Unfortunately, many of the most visible species in cities also tend to be maligned, creating a ‘pigeon paradox’ wherein global conservation is reliant on uncharismatic ambassador animals eliciting charity from urban residents [20]. Future conservation is particularly reliant on urban children, as it is the youngest members of our society who will—in a case of intergenerational inequity—“inherit the earth”, including a litany of environmental problems requiring urgent action [21].

If we aim to promote connectedness with urban nature among all young people, then we must understand how traits such as age and gender affect nature connection and its desired outcome, lifelong pro-environmental behavior. To date, only one longitudinal study has directly investigated the link between people’s environmental behavior in childhood and adulthood (i.e. from age 6 to 18); the former was a poor predictor of the latter [22], with environmental behaviors and attitudes declining throughout middle adolescence [23]. If age does impact pro-environmental behaviors and nature connections in a predictable manner, environmental educators can target interventions so that committed connectedness with nature—and its many attendant benefits—may be maintained throughout childhood and adolescence (and beyond). Likewise, if we understand how gender affects committed connectedness with nature, schools can tailor their environmental education curricula to better meet the needs of their particular student demographics.

In the rare instances where researchers have studied the effects of gender on youth environmentalism, they have typically found that girls report more committed pro-environmental behaviors and stronger connections with nature than boys [21, 24–27]. One Australian study found that gender differences in environmental behavior were minimal, with the authors choosing to statistically control for its effects [28]. This approach is typical; although it appears as a statistical parameter in many models of pro-environmental behavior, researchers usually address gender in passing, with little or no theoretical discussion of its impact [29]. Yet, there is a practical need to explore overlooked effects of gender on children’s and adolescents’ environmental behavior. If, for example, boys are not doing their part for conservation, it may be necessary to target interventions towards strengthening their connections with nature. Furthermore, by understanding how age and gender interact to affect committed connectedness with nature, researchers can better assess how any potential nature deficit is distributed throughout youth populations.

Some previous studies of nature connection in classes of school-aged children and adolescents have shown that younger cohorts are more closely connected with nature than their older peers [30, 31]. When a segmented linear modeling approach was applied on a much broader scale with thousands of 5- to 75-year-old people in England, a single breakpoint in Connection to Nature Index (CNI) scores was identified at age 12 for females and age 11 for males [32]. However, alternative models with two or more breakpoints—illustrating a decline in nature connection during the mid- to late-teenage years—fit that dataset better [32]. This was supported by another cross-sectional English study, which showed that respondents’ connections with nature were weakest between ages 13 and 15 [33]. Taken together, these findings provide evidence that at some point during their late childhood or early adolescent years, young people begin to experience a disconnect from nature. In the absence of longitudinal data, it is unclear as to whether these people reconnect with nature later in life, though cross-sectional trends show a rebound in connectedness throughout adulthood [32, 33], with older adults exhibiting the strongest nature connections [34]. If a decline truly does occur in childhood, it is cause for concern, because young people who are nature-deficient lose the opportunity to benefit both physiologically and psychologically from connecting with nature.

Studies of youth environmentalism have revealed similar age-related patterns, with younger school students undertaking more pro-environmental behaviors than their older peers [35]. In Spain, childhood environmentalism may decline to a minimum around age 15, gradually rebounding in adulthood [36]. This pattern has been described by Swedish researchers as an “adolescent dip” [37]. No such dip was apparent in a smaller study of 12- to 17-year-old Australian students, wherein age was not significantly associated with environmental behavior [28]. Little is known about nature connections in youth populations outside Western cultures [38], so it is currently difficult to ascertain whether age-related trends are similar around the world. It is essential to determine if the adolescent dip is real, because all citizens—not just the youngest ones—must cooperatively act to curb the climate and biodiversity crises. If previously committed citizens “dip out” as they enter adolescence and fail to reconnect with nature, progress towards sustainability and conservation may be unnecessarily threatened.

Only two previous studies of children and adolescents have investigated patterns in both nature connection and environmental behavior with respect to age. The first identified a negative relationship between age and environmental behavior in Spanish children aged 6–12, though this effect was not mediated by emotional affinity with nature [39]. In contrast, a decline in pro-environmental behavior between Canadian students in middle school (age ~12), high school (age ~16), and university (age ~20) was mediated both by nature connection and prescriptiveness of moral judgment [40]. These findings highlight the incomplete state of knowledge regarding the role of nature connection in promoting pro-environmental behavior; though the two constructs are theoretically linked, it is possible that each manifests independently (as in Spain) and must, therefore, be promoted separately. In practice, if nature connection and pro-environmental behavior are closely linked, a single intervention can have a two-for-one impact in fostering positive outcomes, both personal and environmental. Promising intervention strategies include noticing nature [41], multisensory engagement [42], and outdoor learning [30] at Forest Schools [43].

Here, we investigate nature connection and environmental behavior in children and adolescents from Sydney, Australia. We aim to determine the impacts of age and gender on measures of these two related constructs. We hypothesize that nature connection will effectively predict pro-environmental behavior. Accordingly, we further hypothesize that both measures will decline with increasing age, and that girls will report stronger connections with nature and increased pro-environmental behavior compared to boys. Because ours is the first cross-sectional study of nature connection in Australian adolescents [44], we also aim to describe the study population in terms of how strongly they connect with nature. In doing so, we validate the Connection to Nature Index [45] for the first time in an Australian cultural context.

## Materials and methods

### Measures

To ensure we measured students’ cognitive, emotional, and experiential connectedness with nature, we designed a questionnaire that included a multi-dimensional index of ‘connection to nature’ *sensu stricto* [45], as well as a range of questions addressing environmental behaviors accessible to children and adolescents. We also collected demographic information—including age, gender, school, and year of study—from respondents. Items within each index were randomly ordered by the online questionnaire platform ‘Surveymonkey’.

The Connection to Nature Index (CNI) is a trait measure of nature connection intended for use with children [40]. The CNI contains 16 items rated on a 5-point Likert scale from *strongly disagree* to *strongly agree*. The index includes four subscales labeled ‘enjoyment of nature’, ‘empathy for creatures’, ‘sense of oneness’, and ‘sense of responsibility’. A score is calculated by taking the mean of all 16 items; values above 4.56 are considered strong and those below 4.06, weak [26]. These thresholds were previously benchmarked against probabilities of undertaking pro-environmental and pro-nature behaviors, setting them apart from arbitrary norm-based boundaries [26]. In this study, the CNI had high overall internal reliability (Cronbach’s *α* = 0.88).

As a measure of everyday environmental behaviors, we asked participants to indicate how often they made an effort to conserve water, conserve energy, and recycle rubbish on a 5-point Likert scale from *never* to *always*. This cluster of items had moderate internal reliability (Cronbach’s *α* = 0.76), reflecting relatively high levels of engagement in recycling behaviors [33]. We also asked participants if they were willing to volunteer to help protect nature, donate money to nature charities, and talk with friends and family about protecting nature. They responded on a 4-point Likert scale from *strongly disagree* to *strongly agree*. This trio of items had high internal reliability (Cronbach’s *α* = 0.83).

### Participants and procedures

We distributed our online questionnaire to 16 public (i.e. solely government-funded) schools located throughout Sydney, Australia. Eight of these were primary schools with classes from kindergarten through grade 6 (ages ~5–11); eight were secondary schools with students in grades 7–12 (ages ~12–18). All but one of the schools was co-educational; an all-girls’ secondary school was also included in our sample. Socioeconomic disadvantage, as measured by the Family Occupation and Education Index [46], was distributed in a similar manner across participating schools (*M* = 95, *SD* = 54) [47] and public schools (*M* = 100, *SD* = 50) throughout the state of New South Wales (NSW) [48], *t*(2180) = 0.40, *p* = 0.69. The proportion of students at participating schools with a language background other than English (LBOTE) ranged from 9% to 97% [49]. Our median value of 44% accords well with the proportion of public school students in Sydney (53%) and NSW (34%) reporting a LBOTE when the survey was administered [48]. Likewise, the proportion of forested area within the catchment of each participating school (0–55%, $\tilde{X}$ = 12%) corresponded to estimates of canopy cover for Sydney’s Local Government Areas (LGAs), which ranged between 12% and 59% [50]. One in three LGAs had more than 30% canopy cover [50], as did approximately one third (5/16) of catchments for our participating schools. One quarter of participating schools were also located in catchments adjoining coastal water bodies, meaning students attending those institutions lived beside expansive blue spaces. By contrast, one in four participating schools was located more than 40km inland. Together, these metrics indicate that participating institutions are demographically and geographically representative of public schools in Sydney (and NSW more broadly).

We recruited respondents opportunistically across grades 3–8 (ages ~8–14), with a goal of at least 50 students per year group. This would confer sufficient power to detect a small to moderate effect of age (effect size *f* ≈ 0.2). Participants in secondary school provided informed consent, whereas primary schoolers assented with additional written consent from a parent or carer. Ethical protocols were approved via both the University of Sydney’s Human Research Ethics Committee (#2016/961) and the New South Wales Government’s State Education Research Applications Process (#2016467).

Classroom teachers were responsible for distributing participant information statements and consent forms to all their students. They also administered the questionnaire in March of 2017, under controlled conditions such that students did not research their answers. A total of 1,269 questionnaires were returned, including 1,157 that were more than 90% complete. Of these, 1,037 were from respondents who specified both their age and gender, forming the dataset analyzed here (see S1 Table for a demographic breakdown).

### Data analyses

For the 16 CNI items, we assigned each answer an integer representing its position on a 5-point Likert scale (i.e. *strongly disagree* scored 1 of 5 points). Environmental behavior items were encoded likewise. We then imported these ordinal numeric data—along with grouping variables “age” (range: 8–14), “gender” (boy/girl/rather not say), and “ID” (range: 1–1,037)—into R version 3.5.0 [51] for visualization with ‘ggplot2’ [52] and analysis in RStudio version 1.1.453 [53]. In the rare and random cases where data were missing, we imputed values using proportional odds logistic regression via the ‘mice’ and ‘complete’ functions [54].

We calculated an overall CNI score by taking the mean of all 16 items on the index [26]. We also converted data from our environmental behavior items to binary responses: for frequency of conserving energy, conserving water, and recycling, the *always* category scored 1 and all other options 0; for willingness to volunteer, donate money, and advocate for nature, *agree* and *strongly agree* scored 1 whereas *disagree* and *strongly disagree* scored 0. We chose to collapse the categories in this way because we are primarily interested in committed connectedness with nature; making an effort to always undertake pro-environmental behaviors demonstrates commitment.

In order to determine the CNI’s structure using exploratory factor analysis (EFA), we first produced a polychoric correlation matrix of the 16 CNI items using the ‘lavCor’ function [55]. We generated eigenvalues from the correlation matrix with the ‘eigen’ function, subsequently using them in a ‘parallel’ analysis to determine the optimal number of factors to extract via ‘plotnScree’ [56]. In this case, a three-factor structure was optimal (S1 Fig). Because the CNI is typically treated as a unidimensional measure with some internal structure—i.e. subscales—we utilized the ‘omega’ function to implement a bifactor EFA with minimal residual factor analysis, oblimin rotation, and Schmid-Leiman solutions [57]. We grouped CNI items based on their unique Schmid-Leiman factor loading scores > 0.2 [57] and thus generated a bifactor model in ‘lavaan’ syntax for further testing [55].

The three grouping factors identified in our EFA (S2 Fig) differed somewhat from the four subscales developed by the index’s originators [45]. Whereas our first two specific factors corresponded to the ‘enjoyment of nature’ and ‘empathy for creatures’ subscales, the third combined both the ‘sense of oneness’ and ‘sense of responsibility’ subscales of the CNI (thus, we have labeled it ‘sense of oneness & responsibility’) (S2 Table). The questionnaire item “Being outdoors makes me happy” loaded on the ‘enjoyment of nature’ scale; we did not observe a cross-loading (> 0.2) with ‘sense of oneness’ as is normally assumed when researchers score the CNI subscales (S2 Table).

To assess how well the bifactor model fit our sample, we conducted two confirmatory factor analyses (CFAs)—one for girls and another for boys—with theta parameterization, diagonally weighted least squares (WLSMV) estimators, and the fixed-factor method of scale identification; using the ‘cfa’ function [55]. We selected the scaled Comparative Fit Index (CFI), scaled root mean squared error of approximation (RMSEA), Standardized Root Mean Residual (SRMR), and scaled chi-square value as measures of fit [58]. We then used the ‘measEq.syntax’ function [59] with the aforementioned CFA specifications to generate syntax for conducting an equivalent test of model fit across both gender groups, allowing us to assess measurement invariance at the configural level [*sensu* 60]. The syntax was, once again, run using the ‘cfa’ function. This measurement invariance testing process was repeated to fit increasingly restrictive nested models that constrained the following item parameters in sequence: thresholds (“threshold” model), thresholds & loadings (“metric” model), then thresholds & loadings & intercepts (“scalar” model) [61]. Because our scalar model followed a bifactor structure, we manually freed the means of latent variables as advised in the ‘measEq.syntax’ source code [59]. We compared each model’s fit with its parent’s using a scaled chi-squared test [58] in the ‘lavTestLRT’ function [55].

When a significant chi-square test statistic revealed noninvariance between “boy” and “girl” groups at the scalar level, we used the ‘miPowerFit’ function to identify the questionnaire item that was most noninvariant [59]. We then freed the item intercept, creating a partially invariant scalar model that was re-tested against the metric model within which the fully invariant scalar model was originally nested. This process was repeated on items tagged as misspecified by ‘miPowerFit’ until the partially invariant scalar model fit our data as well as the metric model.

Applying ‘lavInspect’ and ‘lavPredict’ functions to the final partially invariant model, we estimated values for the latent variables underlying each respondent’s CNI scores using the Empirical Bayes Modal (EBM) method with Broyden-Fletcher-Goldfarb-Shanno (BFGS) optimization [55]. We then appended these “factor scores” for ‘connection to nature’, ‘enjoyment of nature’, ‘empathy for creatures’, and ‘sense of oneness & responsibility’ to the original dataset.

We tested the effects of age and gender—and the interaction between these two predictors—on latent variables by defining models using the ‘art’ function and running a corresponding ‘ANOVA’ on aligned rank-transformed factor scores for each model [62]. We selected this statistical testing procedure because it was specifically designed as a nonparametric equivalent to a two-way analysis of variance, suitable for testing skewed and otherwise abnormally-distributed data typically derived from Likert scales [62]. Outputs include partial eta-squared (η_p_^2^) values for effect sizes. Where effects were statistically significant, we used the functions ‘emmeans’ and ‘contrast’ to conduct pairwise post-hoc tests that indicated the location and valence of differences [63]. This analysis was also repeated on raw CNI scores.

We evaluated the effects of age, gender, and CNI score on respondents’ environmental behaviors using binary logistic regression via the ‘glm’ function [51]. Goodness-of-fit for each model was assessed using Hosmer-Lemeshow tests returned by the ‘logitgof’ function [64].

## Results

### Connection to nature index

**Fig 1:**
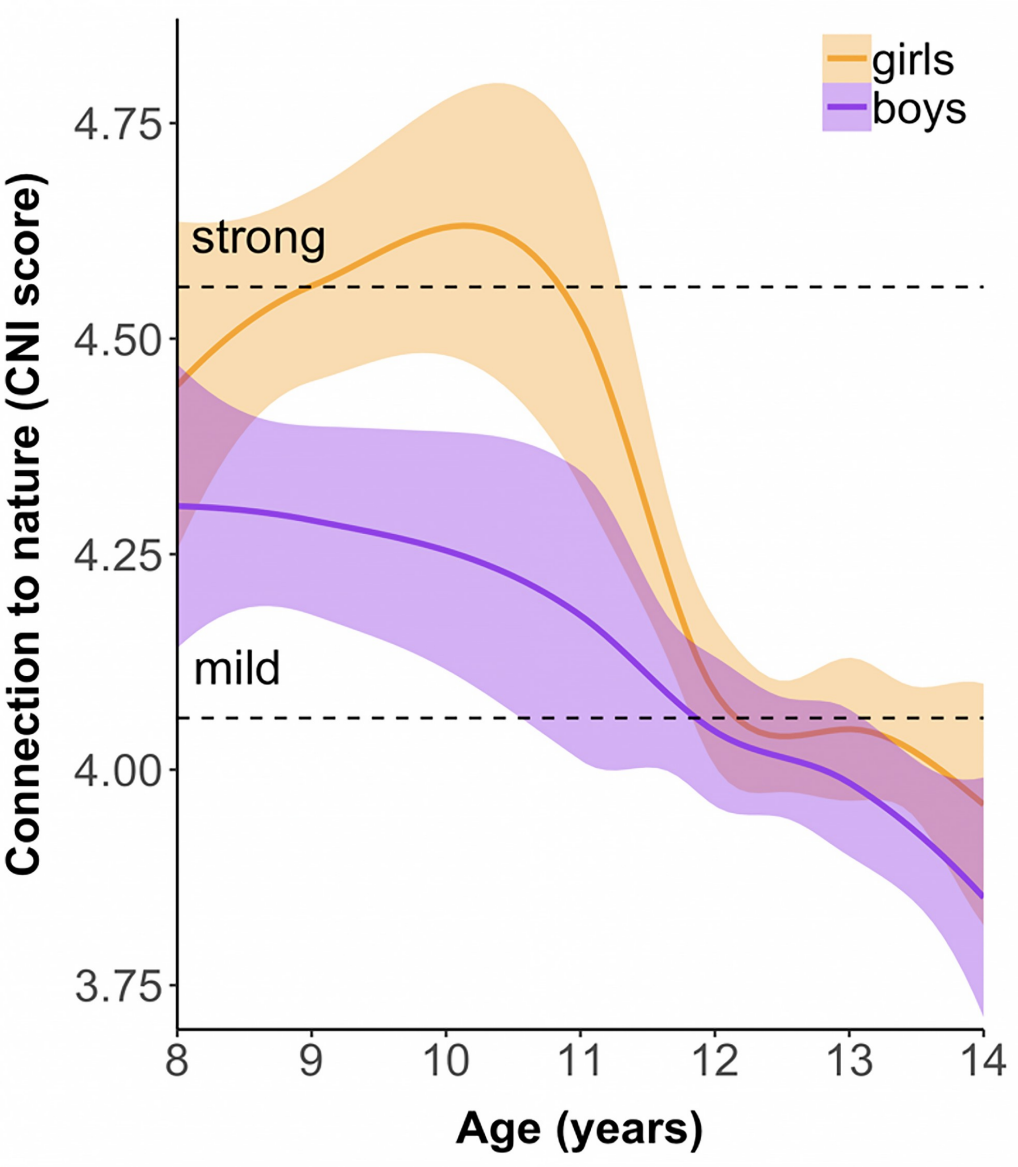
Connection to nature across middle childhood and adolescent years. Trend lines are smoothed Loess curves showing conditional means with 95% CI shaded. *N* = 1,037 (569 girls and 468 boys); see S1 Table for a breakdown by age.

Respondents’ nature connections (Fig 1) were affected by age, *F*(6, 1023) = 17.83, *p* < 0.001, η_p_^2^ = 0.09. They also differed between gender groups, *F*(1, 1023) = 6.98, *p* = 0.008, η_p_^2^ = 0.01. However, there was no significant interaction between terms, indicating gender bias in sampling did not account for observed age effects, *F*(6, 1023) = 1.23, *p* = 0.29, η_p_^2^ = 0.001. Girls (*M* = 4.17 ± 0.05, *n* = 569) scored higher than boys (*M* = 4.06 ± 0.05, *n* = 468) on the CNI (S3 Table), and children aged 8–11 scored higher than adolescents aged 12–14 (S4 and S5 Tables).

Overall, 290 (28%) respondents attained CNI scores indicative of a strong connection with nature (CNI > 4.56), 317 (31%) demonstrated a moderate connection (4.06 < CNI < 4.56), and the remaining 430 (41%) were weakly connected (CNI < 4.06). Proportions differed between genders, with a 34% - 28% - 38% split for girls and a 21% - 33% - 46% spread for boys (S3 and S4 Figs).

The standard four-subscale model of the CNI fit our dataset very poorly, χ^2^ (103, *N* = 1,037) = 6,386.50, CFI = 0.52, RMSEA = 0.24, SRMR = 0.31. Our bifactor model fit much better, χ^2^ (88, *N* = 1,037) = 417.50, CFI = 0.98, RMSEA = 0.06, SRMR = 0.03. The fit was very good for boys’ responses, χ^2^ (88, *N* = 468) = 226.89, CFI = 0.97, RMSEA = 0.06, SRMR = 0.04. It was similar for girls’ responses, χ^2^ (88, *N* = 569) = 304.70, CFI = 0.98, RMSEA = 0.07, SRMR = 0.04.

In our bifactor model, each item on the CNI measured one common construct—i.e. connectedness with nature—in addition to one of three specific factors: ‘enjoyment of nature’, ‘empathy for creatures’, and ‘sense of oneness & responsibility’ (S2 Fig, S2 Table). However, the presence of multidimensionality was not severe enough to disqualify interpretation of the CNI as a primarily unidimensional measure of nature connection (as PUC = 0.70, ECV = 0.66, Ω_H_ = 0.77) [65].

We detected scalar noninvariance of CNI responses across gender groups (Table 1), meaning significant differences between boys’ and girls’ scores could be driven by measurement bias. It was apparent that boys and girls differed in their interpretation of the following four questionnaire items: “I like to hear different sounds in nature”, “collecting rocks and shells is fun”, “I like to see wild flowers in nature”, and “I enjoy touching animals and plants” (S6–S9 Tables).

**Table 1:** Fit indices for multigroup confirmatory factor analyses of items on the connection to nature index.

Model	Overall Fit Indices					Comparative Fit Indices					P- value
	χ^2^	*df*	CFI	RMSEA	SRMR	|Δχ^2^|	|Δ*df*|	|ΔCFI|	|ΔRMSEA|	|ΔSRMR|	
Configural	517.34	176	0.976	0.061	0.040						
Threshold	554.13	208	0.976	0.057	0.040	28.82	32	0.000	0.005	0.000	0.629
Metric	527.18	236	0.980	0.049	0.043	37.40	28	0.004	0.008	0.003	0.110
Scalar	582.53	248	0.977	0.051	0.043	50.74	12	0.003	0.002	0.000	< 0.001
Scalar I	554.85	247	0.978	0.049	0.044	33.18	11	0.001	0.000	0.001	< 0.001
Scalar II	544.02	246	0.979	0.048	0.043	25.55	10	0.000	0.000	0.001	0.004
Scalar III	532.21	245	0.980	0.048	0.043	17.23	9	0.000	0.001	0.000	0.045
Scalar IV	522.15	244	0.981	0.047	0.043	10.01	8	0.001	0.002	0.000	0.264

Measurement invariance was evident for thresholds (‘threshold’ model) and loadings (‘metric’ model) but not intercepts (‘scalar’ model), necessitating establishment of a partially invariant model by freeing intercepts of items “I like to hear different sounds in nature” (scalar I), “Collecting rocks and shells is fun” (scalar II), “I like to see wild flowers in nature” (scalar III), and “I enjoy touching animals and plants” (scalar IV) in a stepwise manner. Comparative fit indices were calculated between consecutively listed models, except for scalar I-IV, which were all compared to the metric model.

### Specific components of nature connection

**Fig 2:**
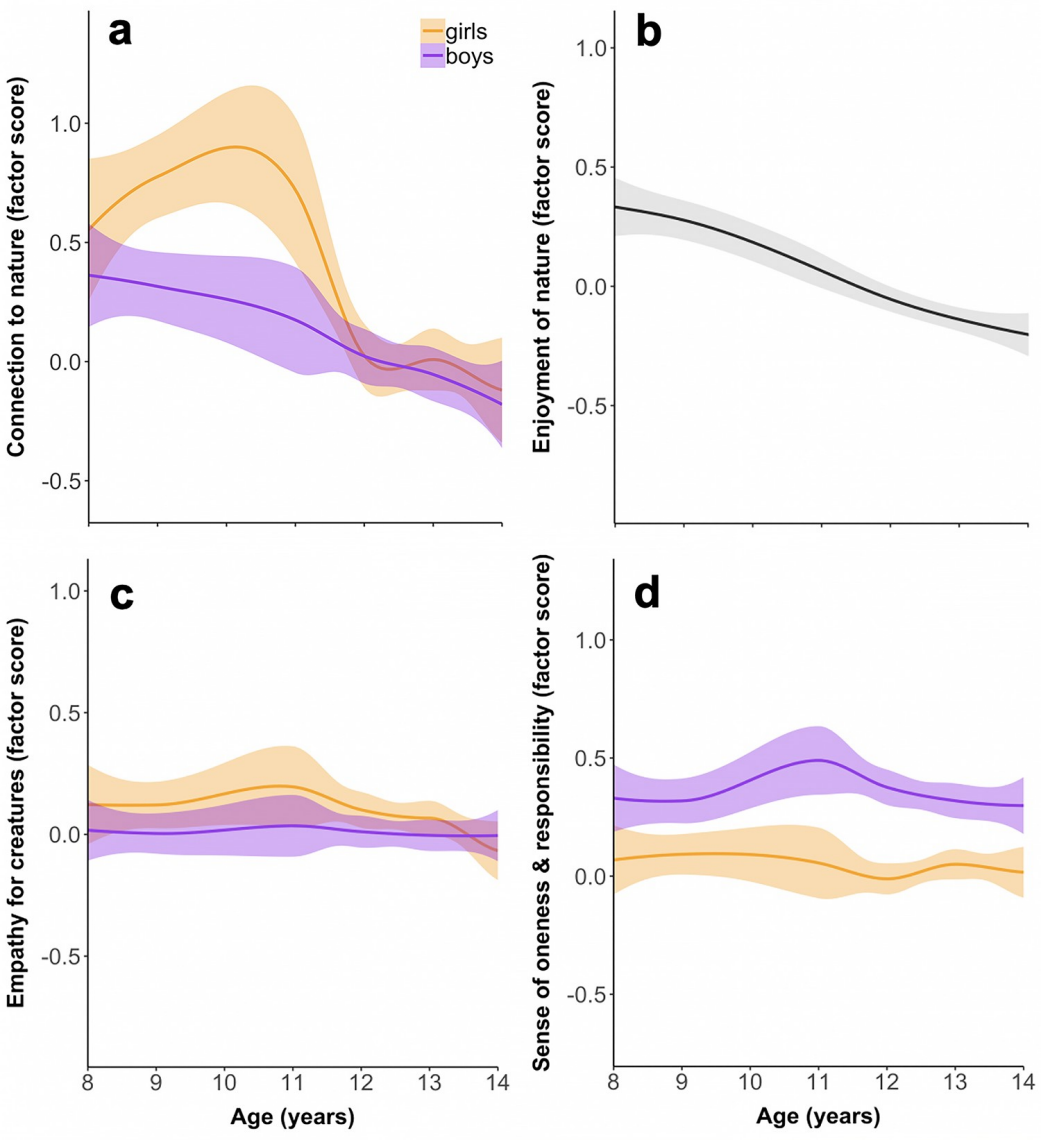
Factor scores for (a) connection to nature and (b–d) its component measures, across middle childhood and adolescent years. Boys and girls were pooled for visualization in panel b because gender did not significantly impact enjoyment of nature. Trend lines are smoothed Loess curves showing conditional means with 95% CI shaded. *N* = 1,037 (569 girls and 468 boys); see S1 Table for a breakdown by age.

When we extracted and analyzed factor scores from a model that addressed scalar noninvariance (by freeing intercepts of the four aforementioned items), the effect of gender on nature connection was no longer significant, *F*(1, 1023) = 3.37, *p* = 0.07, η_p_^2^ = 0.003. The main effect of age remained, *F*(6, 1023) = 15.36, *p* < 0.001, η_p_^2^ = 0.08 (S10 and S11 Tables). The age-by-gender interaction was also significant, *F*(6, 1023) = 2.54, *p* = 0.02, η_p_^2^ = 0.01. In cohorts of children aged 10–11, girls were more closely connected with nature than boys (Fig 2A), but there was no substantive difference between boys’ and girls’ scores in other age groups (S12 and S13 Tables).

Whereas ‘empathy for creatures’ and ‘sense of oneness & responsibility’ were constant across age groups (Fig 2C and 2D), age-based cohorts did differ in their ‘enjoyment of nature’ (Fig 2B), *F*(6, 1023) = 14.26, *p* < 0.001, η_p_^2^ = 0.08. Children aged 8–10 scored higher than adolescents aged 12–14 (S14 and S15 Tables). Across all ages, girls demonstrated more ‘empathy for creatures’ than boys did (S16 Table), *F*(1, 1023) = 5.70, *p* = 0.02, η_p_^2^ = 0.01. Boys reported a stronger ‘sense of oneness & responsibility’ than girls did (S17 Table), *F*(1, 1023) = 82.52, *p* < 0.001, η_p_^2^ = 0.07.

### Environmental behaviors

**Fig 3:**
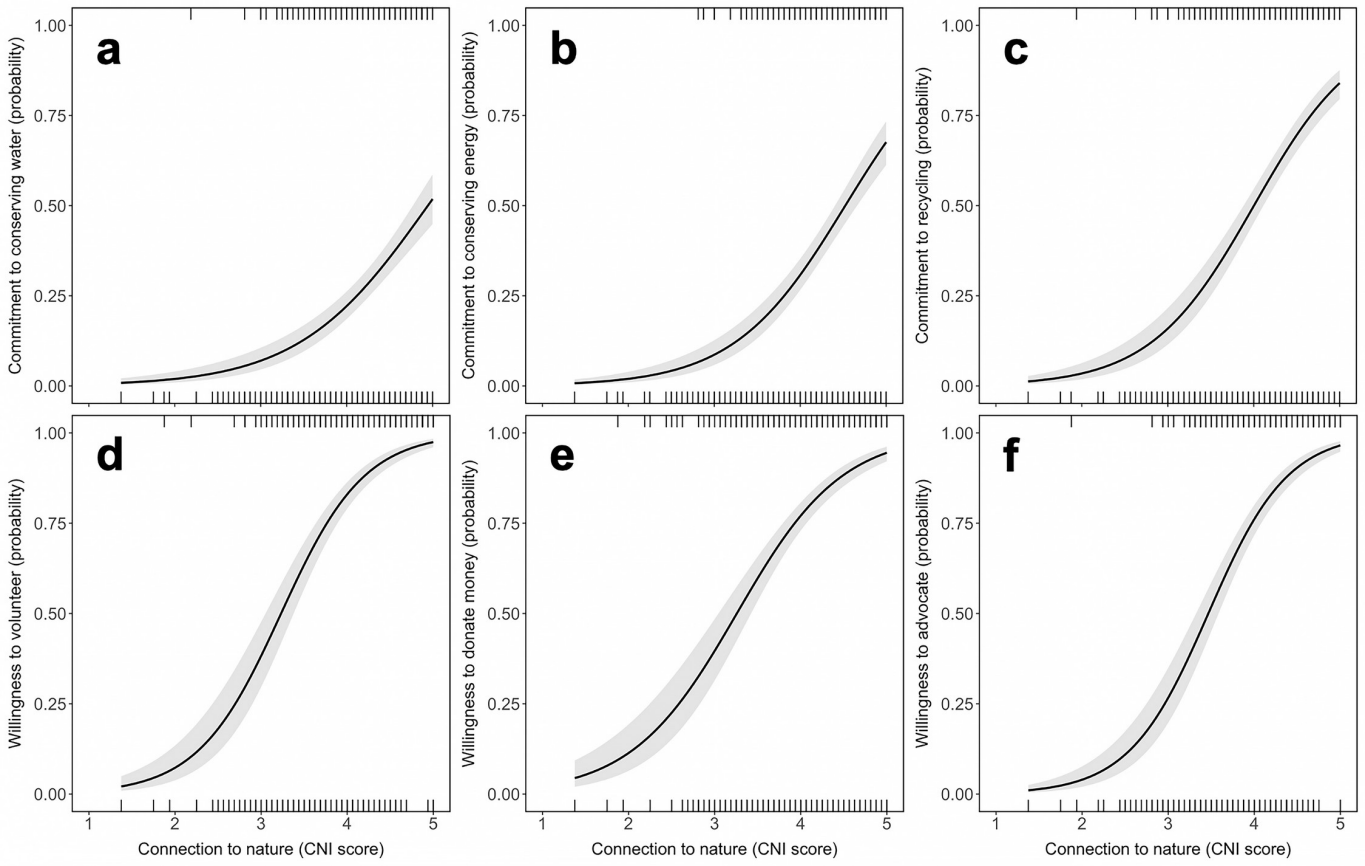
Probability indicating (a–c) commitment to environmental behaviors and (d–f) willingness to conserve nature, graphed as a function of Connection to Nature Index scores. Trend lines show conditional means with 95% CI shaded. Rug lines denote the positions of positive and negative residuals. *N* = 1,037.

Dichotomous logistic regression models—including CNI score, age, and gender as predictors—fit our binomial data on everyday environmental behaviors and willingness to conserve nature well (S18 Table), 6.30 ≥ χ^2^ (3, *N* = 1,037) ≥ 0.87, 0.10 ≤ *p* ≤ 0.83. With each half point increase in CNI score, the odds of always making an effort to conserve water, conserve energy, or recycle waste increased by 94%, 117%, and 130% respectively (Fig 3A–3C); the odds of indicating a willingness to volunteer, give money to nature charities, or talk with friends and family about conserving nature increased by 181%, 126%, and 196% (Fig 3D–3F), holding age and gender constant (S19 Table).

**Fig 4:**
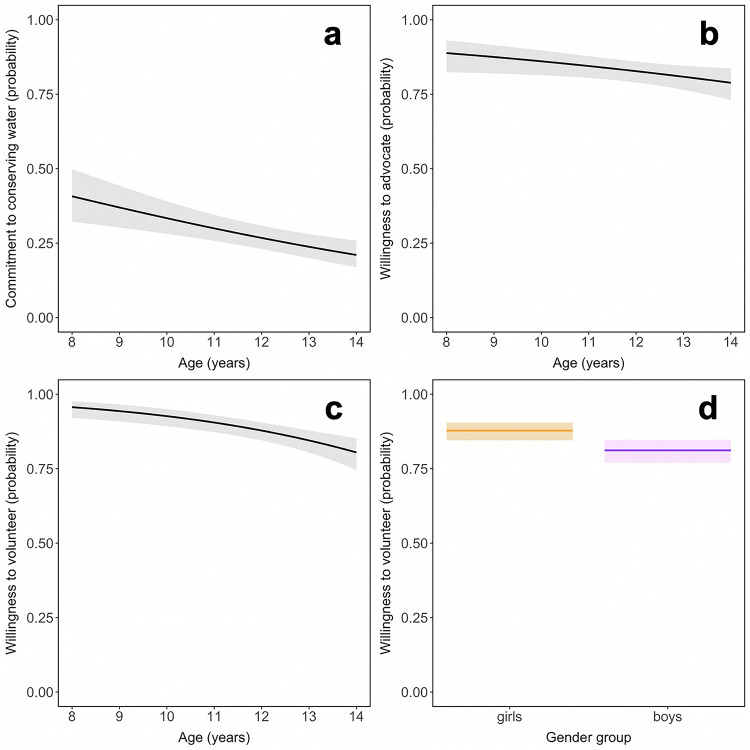
Behavioral outcomes graphed as a function of (a–c) age and (d) gender, independent of connectedness with nature. Trend lines show conditional means with 95% CI shaded. *N* = 1,037 (569 girls and 468 boys).

Irrespective of gender and CNI score, with each increasing year of age, the odds of always making an effort to conserve water declined by 15% (S19 Table), as did the odds of indicating willingness to volunteer or advocate for nature conservation (by 24% and 12% per year, respectively) (Fig 4A–4C). Irrespective of age and CNI score, the odds of being willing to volunteer for conservation were 40% lower for boys than for girls (Fig 4D).

## Discussion

Consistent with our hypothesis that nature connection would decline with age, we found a consistent pattern across the childhood and adolescent years: 8- to 11-year-olds were more closely connected with nature than 12- to 14-year-olds. These two age groups differed in terms of affective connectedness with nature and—perhaps consequently—their efforts to enact pro-environmental behaviors. As hypothesized, nature connection was a strong predictor of behavioral commitment. It was evident that girls generally formed closer emotional connections with nature than boys did, though this apparent trend was complicated once we accounted for respondents’ differential interpretation of CNI items. Regardless, girls were more willing than boys to conserve nature by volunteering. These findings provide partial support for our hypothesis that girls’ behavioral commitment and nature connections would be stronger than boys’. By exploring these emergent patterns in finer detail, we can better understand the association between demographics and committed connectedness with nature.

While previous studies typically combined participant ages into coarse groups or modeled age across decades, we investigated age-related patterns of nature connection and environmental behavior for individual year groups in childhood and adolescence. This allowed us to more precisely identify a transition point between ages 11 and 12 that demarcates two groups: nature-connected primary schoolers and relatively disconnected students in secondary schools. Instead of finding a gradual “adolescent dip” in environmental behavior and nature connection [37], we revealed an abrupt drop—most obviously for girls—around the age at which Australian students move between schools, peer groups, and curricula when transitioning from primary to secondary education. It is also the age at which many children transition to adolescence.

Associations between age and pro-environmental behavior or nature connection are typically interpreted as either true longitudinal effects or generational effects [32, 66]. The clustering of 8- to 11- and 12- to 14-year-olds in this study does not resemble a generational effect, because those typically manifest as gradual changes in attributes across several years [32, 66]. Considering the difference we identified was between students who were separated by 1 year (or less) in age, we suggest that another type of cohort effect—one reflecting the stage of schooling—interacts with a true longitudinal effect (i.e. adolescence) to distinguish between the nature connections and pro-environmental behaviors of the two age groups within our study population. This implies that primary schoolers become increasingly disconnected and apathetic towards pro-environmental behaviors if an impactful intervention is not implemented around the time children are moving on to secondary school.

Preadolescence is recognized as an important time of change in a child’s social development, in which self-conceptions are transformed alongside family and peer relations [31, 66]. The association of a significant educational milestone with the age at which most children begin to transition into adolescence can catalyze this transformation [67]. The need to establish peer relationships at a new school or campus could conceivably force young adolescents to rapidly re-evaluate their self-concept in the context of an expanding social reference group, leading to change [68]. As environmental self-identity—which is conceptually intertwined with nature connection [69]—is a component of a child’s self-concept, we would expect it to also change at this time [70]. Thus, the early adolescent years are strong candidates for implementing interventions that improve connectedness, such as creating artworks inspired by nature [71], or engaging with simple nature-focused activities like bird watching [72].

The transition from primary to secondary schooling is associated with increasing autonomy for young people, who are seen to be “growing up” when they graduate from one school to another. This is often linked to changes in parental licensing, as caregivers allow their offspring to undertake new activities—e.g. traveling to school alone—by virtue of their perceived maturity [73]. However, an increase in independence may lead to emotional autonomy and a concomitant decline in a young adolescent’s feelings of connectedness to their caregiver [31]. If nature is considered a relationship partner [74]—as theorists assert it should be [75]—then nature connection would be expected to decline as emotional autonomy increases in adolescence [31]. This is consistent with the postulate that young children have a somewhat innate sense of relatedness to the natural world, which is later eroded by socialization and maturation [76].

Changes in connectedness with nature might also be driven by biological factors. As the infant brain matures, it functions in less entropic (i.e. more ordered) states [77], but heightened entropy—which is common throughout infancy—induces a subjective feeling of connection with nature [78]. Hence, a loss of entropy with age may cause a reduction in the strength of nature connection. So too could the onset of puberty, which causes developmental changes in arousal, motivation, and emotion [79]. This would account for some of the shifts in priorities described by teenagers whose enthusiasm for environmentalism waned upon entering secondary school, for example, “as I’ve gotten older, other things have become important as well, like boys and clothes and, you know, that kind of thing” [80]. Taken together, this line of reasoning provides a theoretical basis for the difference in affective connectedness with nature between the younger and older students in our study. Going forward, it is essential to test this theory further by determining how important each potential driver of nature disconnection can be. This will help us understand if an “adolescent dip” is, for example, more an unavoidable result of ontogenetic shifts in brain chemistry than it is a relatively malleable outcome of socialization in different academic environments. Cross-cultural studies, in particular, will enable researchers to distinguish between the aforementioned catalysts of change.

Overall, connectedness with nature was a consistently strong predictor of everyday environmental behaviors and willingness to conserve nature in this study. However, it is possible for age to impact behavior independently [39], via developmental effects that are not tied to nature connection. As children grow into adolescents, peers become more influential in motivating their behaviors [25]. This has been demonstrated for 9- to 13-year-old students, with the environmental behavior of best friends having a greater impact as age increases [25]. Here, we show that with each increasing year of age, respondents were progressively less eager to talk with their friends (and family) about nature conservation. It is unclear whether this is a cause or a symptom of the parallel decline in environmental behaviors (e.g. efforts to conserve water) we also detected, but—in any case—the co-occurrence of these effects is expected where conformity to social norms is important [39].

The difference in environmental behavior between our primary and secondary school cohorts may reflect a decline in the relative importance of pro-environmentalism among social groups in secondary school, where students tend to be more interested in alternative developmentally linked activities (e.g. studying, dating, competitive sports). Although social conformity was the least—and laziness the most—common reason given by Australian adolescents in a previous study to explain their disengagement with pro-environmental behaviors [21], one in three of those respondents still indicated they did not have enough time to act pro-environmentally [21], potentially reflecting an opportunity cost of engaging in other activities. This presents a challenge to environmental educators working in secondary schools: how might we best encourage adolescents to undertake pro-environmental behaviors, given the many competing demands on their time and paradoxical predisposition to laziness? Do girls—whose nature connections seem to differ most between primary and secondary groups—disconnect from nature more than boys in early adolescence, or do boys just not have as far to fall?

Across our study population, we found that girls were more closely connected with nature and more willing to volunteer for conservation projects than were boys. Though this finding is consistent with previous research [29], it does warrant additional explanation. Environmentalism and conservation involve caring for and nurturing nature, which are considered “prototypical” feminine traits [81]. This common perception demonstrates a gender expectation within the context of societal norms [27]. Most human societies engage in differential gender socialization wherein girls and boys are treated differently and encouraged to engage with different activities and ideas [82]. Females are usually socialized to be caring, nurturing, empathetic, compassionate, considerate, and altruistic; on the other hand, males are socialized to be competitive and independent [27, 81, 82]. If girls in our sample have internalized this socialization, it may explain why they scored higher than boys on the ‘empathy for creatures’ component of the CNI. Likewise, if the boys in this study have internalized the societal expectation that they be more self-confident, it might explain why they scored higher than girls on ‘sense of oneness & responsibility’, which includes items related to self-efficacy (e.g. “my actions will make the natural world different”). Self-efficacy can indeed manifest differently in boys and girls, influencing their environmental behaviors [83]. Accordingly, future research interrogating disparities between boys’ and girls’ nature connections should incorporate child-friendly indices to measure and account for how young respondents’ socialized personality traits influence their connectedness with nature.

Our modeling demonstrates that the items “I like to see wild flowers in nature”, “I like to hear different sounds in nature”, “I enjoy touching animals and plants”, and “collecting rocks and shells is fun” drove the statistical difference between boys’ and girls’ CNI scores. The first three of these describe sensory engagement with nature in visual, aural, and tactile terms [7, 72]; and the detection of scalar noninvariance in this cluster of items indicates that boys and girls interpreted them differently. A promising explanation for this discrepancy in interpretation may lie in differing ‘motivation for sensory pleasure’ between gender groups [84]. If the girls in our study population had a higher baseline level of motivation to derive enjoyment from interacting with nature via sensory stimulation—as women did in previous studies [84, 85]—then it follows logically that they would have responded more positively than boys to questionnaire items reflecting this construct. Indeed, that is what we observed. Similarly, if the girls were compelled to collect seashells more than their male peers [see 86], they would be expected to respond preferentially to the “shells” component of the “rocks and shells” CNI item. Thus, we posit that pre-existing differences between boys’ and girls’ modes of interaction with nature may have primed them to interpret the four aforementioned CNI items in nonequivalent ways, leading to their identification in measurement invariance tests. Investigators who intend to use the CNI for measuring connectedness with nature in heterogeneous groups of children and adolescents should do so with caution; here, we have shown that one quarter of its items demonstrate measurement noninvariance between gender groups, and the traditional four-subscale structure proposed by its originators [45] did not fit our sample population.

In addition to identifying statistically significant differences in nature connection and environmental behavior across demographic groups, we also observed substantial intra-group variability within our sample. Although, overall, we found that younger girls generally connected more strongly with nature than older boys, some of the adolescent boys in this study demonstrated a strong connection to nature, and a number of young girls were weakly connected. Participants spanned the full range of connectedness and behavioral commitment. Therefore, making a blanket statement about any demographic group risks oversimplifying or inadvertently erasing the lived experience of individuals who defy the majority. These participants were not irrelevant outliers and have not been treated as such in our analyses; instead they offer insights into what has caused certain children to, for example, connect with nature where others have not. This would be a promising focus for further investigation, ideally using qualitative methods [87] to derive rich descriptive data.

Diversity of experience was clearly evident within our sample population and therefore within urban children and adolescents more generally. When we classified respondents’ CNI scores into three behaviorally relevant categories—“weak”, “moderate”, and “strong” [26]—the split was approximately 40-30-30. Consequently, we cannot say that the majority of respondents in our sample were either weakly or strongly connected with nature. This may, on face value, appear to be a somewhat surprising result, given the widespread use of labels such as “nature-deficit disorder” [13] when describing the experience of “kids these days” [88]. However, cohorts living in cities can be extremely diverse, and we have shown that a number of them manage to connect with nature despite their urban setting and modern lives. Therefore, it is important to keep the population’s spectrum of nature connections in mind when developing policies that affect all children and adolescents within a certain location or demographic group.

It is possible that some of our respondents with strong nature connections may have already benefited from educational policies promoting nature-based learning. Outdoor learning was added to the Australian national curriculum in the year we conducted this survey [89]. However, state educational authorities had reservations about embracing outdoor learning, and teachers were left to decide their approach and levels of engagement [90]. Nevertheless, opportunities for meaningful learning in nature are presented, for example, by the NSW Geography Syllabus, which emphasises connection to place and mandates outdoor fieldwork [90]. Teachers should be empowered and resourced to pursue these opportunities.

By virtue of this study’s cross-sectional design, we are limited to describing associations between demographic groups, their nature connections, and environmental behaviors without conclusively identifying causal mechanisms. Future studies should interrogate our findings using longitudinal and quasi-experimental designs to determine, for example, why exactly the strength of nature connections can differ so markedly between cohorts grouped by consecutive birth years. They should also extend investigations beyond our urban Australian context to explore the same relationships in other geographical and sociocultural settings, ideally using objective (vs. self-reported) behavioral measures. Diversity can be embraced further still by providing participants with opportunities to identify outside the categorical gender options we listed in this study (i.e. “boy”, “girl”, “rather not say”). Research investigating links between gender and nature connections would benefit from the inclusion of genderqueer perspectives, as they provide insights into the influences non-binary identities might have on engagement with nature and environmentalism.

## Conclusions

Despite the aforementioned limitations, we can confidently report that respondents in late childhood enjoyed nature more than their counterparts in early adolescence, forming the basis of stronger affective connections with nature. Younger cohorts also made more of an effort to always undertake everyday environmental behaviors (e.g. conserving water) and were more willing to conserve nature (e.g. by volunteering) than older groups. This result is expected, given the strong predictive relationship we identified between nature connection and behavioral outcomes. The relationship between gender and nature connection is more equivocal, however, as one measure of nature connection (CNI score) indicated that girls connected more strongly with nature than boys did, whereas another (factor score) indicated that this difference only existed for students aged 10–11. Furthermore, girls’ everyday environmental behaviors did not differ substantially from boys’, though they were more willing to conserve nature by volunteering.

Our key findings should inform the development of new environmental curricula and educational practices. Interventions designed to prevent or remediate declines in nature connection will ideally capitalize on evidence-based pathways to connectedness [7, 42]. For example, tapping the ‘emotion’ pathway by incorporating affective elements in outdoor education programs has resulted in students strengthening their connections with nature [30]. Knowing that girls in this study responded most strongly to items referencing sensory stimulation could inspire educators to utilize this pathway [91], in particular when working with groups of girls (whether that be in classrooms, homes, or the great outdoors). Understanding that there is a decline in nature connection coinciding with students’ transition from primary to secondary schooling may help teachers and parents target environmental education to the early adolescent years. Perhaps most importantly, realizing that relationships between children and nature in the city are highly variable might lead us to resist stereotyping our urban youth.

## Supporting information

S1 Fig Scree plot of eigenvalues from a polychoric correlation matrix of CNI responses, indicating that the optimal number of grouping factors in the dataset is three. (TIF)

S2 Fig Each item on the CNI loaded on the general factor “g” (connection to nature) and one specific factor: Either F1 (enjoyment of nature), F2 (empathy for creatures), or F3 (sense of oneness & responsibility). Paths are drawn where the absolute value of the Schmid-Leiman factor loading exceeds 0.2.
(TIF)

S3 Fig Stacked histogram of CNI scores for girls (*n* = 569) and boys (*n* = 468). (TIF)

S4 Fig Proportion of girls (above, *n* = 569) and boys (below, *n* = 468) responding with each point on the Likert scale to individual items on the CNI. Items are ordered according to where they conventionally appear on the CNI.
(TIF)

S1 Table Demographic breakdown of respondents who identified as a girl or a boy and also specified their age. (XLSX)

S2 Table Schmid-Leiman factor loadings for CNI items, returned by the bifactor EFA. F1 is ‘enjoyment of nature’, F2 is ‘empathy for creatures’, F3 is ‘sense of oneness & responsibility’, and g is the general factor ‘connection to nature’. Values are shaded where they do not exceed 0.2.
(XLSX)

S3 Table Girls scored higher than boys on the CNI. (XLSX)

S4 Table Children aged 8–11 scored higher on the CNI than adolescents aged 12–14. (XLSX)

S5 Table Test statistics for pairwise comparisons between age groups of aligned, rank-transformed CNI scores. (XLSX)

S6 Table Determining the first item intercept that should be freed to establish a partially invariant model of the CNI, using the Saris-Satorra-van der Veld method. Items are numbered according to their order in S2 Table and sorted by modification index score.
(XLSX)

S7 Table Determining the second item intercept that should be freed to establish a partially invariant model of the CNI, using the Saris-Satorra-van der Veld method. Items are numbered according to their order in S2 Table and sorted by modification index score.
(XLSX)

S8 Table Determining the third item intercept that should be freed to establish a partially invariant model of the CNI, using the Saris-Satorra-van der Veld method. Items are numbered according to their order in S2 Table and sorted by modification index score.
(XLSX)

S9 Table Determining the fourth item intercept that should be freed to establish a partially invariant model of the CNI, using the Saris-Satorra-van der Veld method. Items are numbered according to their order in S2 Table and sorted by modification index score.
(XLSX)

S10 Table ‘Connection to nature’ was higher across ages 8–11 as compared to ages 12–14. (XLSX)

S11 Table Test statistics for pairwise comparisons between age groups of aligned, rank-transformed ‘connection to nature’ scores. (XLSX)

S12 Table Boys’ and girls’ ‘connection to nature’ scores appeared to differ less in adolescent cohorts (ages 12–14) than they did in childhood cohorts (ages 8–11). See next for detail.
(XLSX)

S13 Table Difference-of-differences tests interrogating how girls’ and boys’ aligned, rank-transformed ‘connection to nature’ scores differ across paired age groups. Girls’ and boys’ scores diverged most in late childhood cohorts (ages 10–11) and least in adolescence (ages 12–14), with middle childhood year groups (ages 8–9) indistinct from either of these.
(XLSX)

S14 Table Children aged 8–10 had higher ‘enjoyment of nature’ scores than did adolescents aged 12–14. Eleven-year-olds’ scores were intermediate between these two groups.
(XLSX)

S15 Table Test statistics for pairwise comparisons between age groups of aligned, rank-transformed ‘enjoyment of nature’ scores. (XLSX)

S16 Table Girls scored higher than boys for the ‘empathy for creatures’ factor. (XLSX)

S17 Table Boys scored higher than girls for the ‘sense of oneness & responsibility’ factor. (XLSX)

S18 Table Goodness-of-fit for models that include age, gender, and CNI score as predictors of each environmental behavior. A p value greater than 0.05 indicates adequate fit.
(XLSX)

S19 Table Age, gender, and connection to nature (CNI score) predict multiple environmental behaviors. A single asterisk denotes significance at the p < 0.05 level; two asterisks at p < 0.01; three asterisks at p < 0.001.
(XLSX)

## References

[1] Steffen W, Richardson K, Rockström J, Cornell SE, Fetzer I, Bennett EM, et al. Planetary boundaries: Guiding human development on a changing planet. Science. 2015; 347(6223): 736. doi: 10.1126/science.1259855 25592418

[2] Milner-Gulland EJ. Interactions between human behaviour and ecological systems. Philos Trans R Soc B Biol Sci. 2012; 367(1586): 270–8. doi: 10.1098/rstb.2011.0175 22144389 PMC3223800

[3] Swim JK, Clayton S, Howard GS. Human behavioral contributions to climate change: Psychological and contextual drivers. Am Psychol. 2011; 66(4): 251–64. doi: 10.1037/a0023472 21553951

[4] Schultz PW. Conservation means behavior. Conserv Biol. 2011; 25(6): 1080–3. doi: 10.1111/j.1523-1739.2011.01766.x 22070255

[5] Manfredo MJ, Bruskotter JT, Teel TL, Fulton DC, Oishi S, Uskul AK, et al. Revisiting the challenge of intentional value shift: reply to Ives and Fischer. Conserv Biol. 2017; 31(6): 1486–7. doi: 10.1111/cobi.13026 28992363

[6] Whitburn J, Linklater W, Abrahamse W. Meta-analysis of human connection to nature and proenvironmental behavior. Conserv Biol. 2020; 34(1): 180–93. doi: 10.1111/cobi.13381 31251416 PMC7027494

[7] Lumber R, Richardson M, Sheffield D. Beyond knowing nature: Contact, emotion, compassion, meaning, and beauty are pathways to nature connection. PLOS ONE. 2017; 12(5): e0177186. doi: 10.1371/journal.pone.0177186 28486515 PMC5423657

[8] Otto S, Pensini P. Nature-based environmental education of children: Environmental knowledge and connectedness to nature, together, are related to ecological behaviour. Glob Environ Change. 2017; 47: 88–94. doi: 10.1016/j.gloenvcha.2017.09.009

[9] Zylstra MJ, Knight AT, Esler KJ, Le Grange LLL. Connectedness as a core conservation concern: An interdisciplinary review of theory and a call for practice. Springer Sci Rev. 2014; 2(1): 119–43. doi: 10.1007/s40362-014-0021-3

[10] McDonnell MJ, MacGregor-Fors I. The ecological future of cities. Science. 2016; 352(6288): 936–8. doi: 10.1126/science.aaf3630 27199416

[11] Soga M, Gaston KJ. Extinction of experience: The loss of human–nature interactions. Front Ecol Environ. 2016; 14(2): 94–101. doi: 10.1002/fee.1225

[12] Hartig T, Kahn PH. Living in cities, naturally. Science. 2016; 352(6288): 938–40. doi: 10.1126/science.aaf3759 27199417

[13] Louv R. Last child in the woods: Saving our children from nature-deficit disorder. 1st ed. Chapel Hill, NC: Algonquin Books; 2005. 323 p.

[14] Keys EB, Lindsey P, Bradley LK, Werner D, Driscoll E. Residential children’s landscapes: connecting with nature in the backyard. Acta Hortic. 2013; 999: 81–8. doi: 10.17660/ActaHortic.2013.999.10

[15] Soga M, Gaston KJ, Yamaura Y, Kurisu K, Hanaki K. Both direct and vicarious experiences of nature affect children’s willingness to conserve biodiversity. Int J Environ Res Public Health. 2016; 13(6): 529. doi: 10.3390/ijerph13060529 27231925 PMC4923986

[16] Collado S, Corraliza JA, Staats H, Ruiz M. Effect of frequency and mode of contact with nature on children’s self-reported ecological behaviors. J Environ Psychol. 2015; 41: 65–73. doi: 10.1016/j.jenvp.2014.11.001

[17] Newman L, Dale A. Celebrating the mundane: Nature and the built environment. Environ Values. 2013; 22(3): 401–13. doi: 10.3197/096327113X13648087563827

[18] Nisbet EK, Zelenski JM. Underestimating nearby nature: Affective forecasting errors obscure the happy path to sustainability. Psychol Sci. 2011; 22(9): 1101–6. doi: 10.1177/0956797611418527 21828351

[19] Gill T. The benefits of children’s engagement with nature: A systematic literature review. Child Youth Environ. 2014; 24(2): 10–34. doi: 10.7721/chilyoutenvi.24.2.0010

[20] Dunn RR, Gavin MC, Sanchez MC, Solomon JN. The pigeon paradox: Dependence of global conservation on urban nature. Conserv Biol. 2006; 20(6): 1814–6. doi: 10.1111/j.1523-1739.2006.00533.x 17181818

[21] Fielding KS, Head BW. Determinants of young Australians’ environmental actions: the role of responsibility attributions, locus of control, knowledge and attitudes. Environ Educ Res. 2012; 18(2): 171–86. doi: 10.1080/13504622.2011.592936

[22] Evans GW, Otto S, Kaiser FG. Childhood origins of young adult environmental behavior. Psychol Sci. 2018; 29(5): 679–87. doi: 10.1177/0956797617741894 29447064

[23] Otto S, Evans GW, Moon MJ, Kaiser FG. The development of children’s environmental attitude and behavior. Glob Environ Change. 2019; 58: 101947. doi: 10.1016/j.gloenvcha.2019.101947

[24] Bragg R, Wood C, Barton J, Pretty J. Measuring connection to nature in children aged 8–12: A robust methodology for the RSPB. Essex Sustainability Institute and School of Biological Sciences, University of Essex; 2013 p. 1–64. Available: https://www.rspb.org.uk/globalassets/downloads/documents/positions/education/measuring-connection-to-nature-in-children-aged-8—12—methodology.pdf

[25] Collado S, Evans GW, Sorrel MA. The role of parents and best friends in children’s pro-environmentalism: Differences according to age and gender. J Environ Psychol. 2017; 54: 27–37. doi: 10.1016/j.jenvp.2017.09.007

[26] Hughes J, Richardson M, Lumber R. Evaluating connection to nature and the relationship with conservation behaviour in children. J Nat Conserv. 2018; 45: 11–9. doi: 10.1016/j.jnc.2018.07.004

[27] Zelezny LC, Chua P-P, Aldrich C. New ways of thinking about environmentalism: Elaborating on gender differences in environmentalism. J Soc Issues. 2000; 56(3): 443–57. doi: 10.1111/0022-4537.00177

[28] Robinson AC, Downey LA, Ford TC, Lomas JE, Stough C. Green teens: Investigating the role of emotional intelligence in adolescent environmentalism. Personal Individ Differ. 2019; 138: 225–30. doi: 10.1016/j.paid.2018.10.009

[29] Sakellari M, Skanavis C. Environmental behavior and gender: An emerging area of concern for environmental education research. Appl Environ Educ Commun. 2013; 12(2): 77–87. doi: 10.1080/1533015X.2013.820633

[30] Braun T, Dierkes P. Connecting students to nature–how intensity of nature experience and student age influence the success of outdoor education programs. Environ Educ Res. 2017; 23(7): 937–49. doi: 10.1080/13504622.2016.1214866

[31] Liefländer AK, Fröhlich G, Bogner FX, Schultz PW. Promoting connectedness with nature through environmental education. Environ Educ Res. 2013; 19(3): 370–84. doi: 10.1080/13504622.2012.697545

[32] Hughes J, Rogerson M, Barton J, Bragg R. Age and connection to nature: when is engagement critical? Front Ecol Environ. 2019; 17(5): 265–9. doi: 10.1002/fee.2035

[33] Richardson M, Hunt A, Hinds J, Bragg R, Fido D, Petronzi D, et al. A measure of nature connectedness for children and adults: Validation, performance, and insights. Sustainability. 2019; 11(12): 3250. doi: 10.3390/su11123250

[34] Dean JH, Shanahan DF, Bush R, Gaston KJ, Lin BB, Barber E, et al. Is nature relatedness associated with better mental and physical health? Int J Environ Res Public Health. 2018; 15(7): 1371. doi: 10.3390/ijerph15071371 29966307 PMC6069224

[35] Wray-Lake L, Metzger A, Syvertsen AK. Testing multidimensional models of youth civic engagement: Model comparisons, measurement invariance, and age differences. Appl Dev Sci. 2017; 21(4): 266–84. doi: 10.1080/10888691.2016.1205495

[36] Pol E, Castrechini A. ¿Disrupción en la educación para la sostenibilidad? Rev Latinoam Psicol. 2013; 45(3): 335–49. doi: 10.14349/rlp.v45i3.1477

[37] Olsson D, Gericke N. The adolescent dip in students’ sustainability consciousness—Implications for education for sustainable development. J Environ Educ. 2016; 47(1): 35–51. doi: 10.1080/00958964.2015.1075464

[38] Chawla L. Childhood nature connection and constructive hope: A review of research on connecting with nature and coping with environmental loss. People Nat. 2020; 2(3): 619–42. doi: 10.1002/pan3.10128

[39] Collado S, Evans GW, Corraliza JA, Sorrel MA. The role played by age on children’s pro-ecological behaviors: An exploratory analysis. J Environ Psychol. 2015; 44: 85–94. doi: 10.1016/j.jenvp.2015.09.006

[40] Krettenauer T. Pro-environmental behavior and adolescent moral development. J Res Adolesc. 2017; 27(3): 581–93. doi: 10.1111/jora.12300 28776840

[41] Harvey C, Hallam J, Richardson M, Wells R. The good things children notice in nature: An extended framework for reconnecting children with nature. Urban For Urban Green. 2020; 49: 126573. doi: 10.1016/j.ufug.2019.126573

[42] Richardson M, Dobson J, Abson DJ, Lumber R, Hunt A, Young R, et al. Applying the pathways to nature connectedness at a societal scale: a leverage points perspective. Ecosyst People. 2020; 16(1): 387–401. doi: 10.1080/26395916.2020.1844296

[43] Cudworth D, Lumber R. The importance of Forest School and the pathways to nature connection. J Outdoor Environ Educ. 2021; 24(1): 71–85. doi: 10.1007/s42322-021-00074-x

[44] Whitten T, Stevens R, Ructtinger L, Tzoumakis S, Green MJ, Laurens KR, et al. Connection to the natural environment and well-being in middle childhood. Ecopsychology. 2018; 10(4): 270–9. doi: 10.1089/eco.2018.0010

[45] Cheng JC-H, Monroe MC. Connection to nature: Children’s affective attitude toward nature. Environ Behav. 2012; 44(1): 31–49. doi: 10.1177/0013916510385082

[46] Lu L, Rickard K. Family occupation and education index (FOEI) 2013. Centre for Education Statistics and Evaluation; 2014 p. 1–40. Available: https://www.cese.nsw.gov.au//images/stories/PDF/FOEI_Technical_Paper_final_v2.pdf

[47] New South Wales Department of Education. The resource allocation model (RAM) in 2018: Local schools, local decisions. 2019 p. 1–47. Available: https://schoolsequella.det.nsw.edu.au/file/a7d46eff-6040-44d9-9ddb-e800420d172c/1/RAM%202018%20-%20Funding%20Table.pdf

[48] Centre for Education Statistics and Evaluation. Schools and students: 2017 statistical bulletin. CESE Statistical Bulletin. 2018; 7: 1–33. Available: https://education.nsw.gov.au/content/dam/main-education/about-us/educational-data/cese/2017-schools-and-students-statistical-bulletin.pdf

[49] Australian Curriculum, Assessment and Reporting Authority. Find a school. My School. 2017. Available: https://www.myschool.edu.au/

[50] Jacobs B, Mikhailovich N, Delaney C. Benchmarking Australia’s urban tree canopy: An i-Tree assessment, prepared for Horticulture Australia Limited. Institute for Sustainable Futures, University of Technology Sydney; 2014 p. 1–43. Available: http://hdl.handle.net/10453/3750640930.pdf

[51] R Core Team. R: A language and environment for statistical computing. Vienna, Austria: R Foundation for Statistical Computing; 2018. Available: https://www.R-project.org/

[52] Wickham H. ggplot2: Elegant graphics for data analysis. New York, NY: Springer-Verlag; 2009. (Use R!). doi: 10.1007/978-0-387-98141-3

[53] RStudio Team. RStudio: Integrated development for R. Boston, MA: RStudio; 2016. Available: http://www.rstudio.com/

[54] van Buuren S, Groothuis-Oudshoorn K. mice: Multivariate imputation by chained equations in R. J Stat Softw. 2011; 45(1): 1–67. doi: 10.18637/jss.v045.i03

[55] Rosseel Y. lavaan: An R package for structural equation modeling. J Stat Softw. 2012; 48(1): 1–36. doi: 10.18637/jss.v048.i02

[56] Raiche G. nFactors: An R package for parallel analysis and non graphical solutions to the Cattell scree test. 2019. Available: http://cran.r-project.org/package=nFactors

[57] Revelle W. psych: Procedures for psychological, psychometric, and personality research. Evanston, IL: Northwestern University; 2019. Available: https://cran.r-project.org/package=psych

[58] Satorra A. Scaled and adjusted restricted tests in multi-sample analysis of moment structures. In: Heijmans RDH, Pollock DSG, Satorra A, editors. Innovations in multivariate statistical analysis: A festschrift for Heinz Neudecker. Boston, MA: Springer; 2000. p. 233–47. (Advanced studies in theoretical and applied econometrics). doi: 10.1007/978-1-4615-4603-0_17

[59] Jorgensen TD, Pornprasermanit S, Schoemann AM, Rosseel Y. semTools: Useful tools for structural equation modeling. 2019. Available: https://cran.r-project.org/package=semTools

[60] Boeve-de Pauw J, Jacobs K, Van Petegem P. Gender differences in environmental values: An issue of measurement? Environ Behav. 2014; 46(3): 373–97. doi: 10.1177/0013916512460761

[61] Wu H, Estabrook R. Identification of confirmatory factor analysis models of different levels of invariance for ordered categorical outcomes. Psychometrika. 2016; 81(4): 1014–45. doi: 10.1007/s11336-016-9506-0 27402166 PMC5458787

[62] Wobbrock JO, Findlater L, Gergle D, Higgins JJ. The aligned rank transform for nonparametric factorial analyses using only ANOVA procedures. In: Proceedings of the 2011 annual conference on human factors in computing systems. Vancouver, BC, Canada: ACM Press; 2011. p. 143–6. doi: 10.1145/1978942.1978963

[63] Lenth R. emmeans: Estimated marginal means, a.k.a. least-squares means. 2019. Available: https://cran.r-project.org/package=emmeans

[64] Jay M. generalhoslem: Goodness of fit tests for logistic regression models. 2019. Available: https://cran.r-project.org/package=generalhoslem

[65] Reise SP, Bonifay WE, Haviland MG. Scoring and modeling psychological measures in the presence of multidimensionality. J Pers Assess. 2013; 95(2): 129–40. doi: 10.1080/00223891.2012.725437 23030794

[66] Soga M, Gaston KJ, Kubo T. Cross-generational decline in childhood experiences of neighborhood flowering plants in Japan. Landsc Urban Plan. 2018; 174: 55–62. doi: 10.1016/j.landurbplan.2018.02.009

[67] Simmons RG, Blyth DA. Moving into adolescence: the impact of pubertal change and school context. 1st ed. Hawthorne, NY: Aldine de Gruyter; 1987. 441 p. (Social institutions and social change). doi: 10.4324/9781315124841

[68] Benner AD. The transition to high school: Current knowledge, future directions. Educ Psychol Rev. 2011; 23(3): 299–328. doi: 10.1007/s10648-011-9152-0 21966178 PMC3182155

[69] Lokhorst AM, Hoon C, le Rutte R, de Snoo G. There is an I in nature: The crucial role of the self in nature conservation. Land Use Policy. 2014; 39: 121–6. doi: 10.1016/j.landusepol.2014.03.005

[70] Cole DA, Maxwell SE, Martin JM, Peeke LG, Seroczynski AD, Tram JM, et al. The development of multiple domains of child and adolescent self-concept: A cohort sequential longitudinal design. Child Dev. 2001; 72(6): 1723–46. doi: 10.1111/1467-8624.00375 11768142

[71] Bruni CM, Winter PL, Schultz PW, Omoto AM, Tabanico JJ. Getting to know nature: evaluating the effects of the Get to Know Program on children’s connectedness with nature. Environ Educ Res. 2017; 23(1): 43–62. doi: 10.1080/13504622.2015.1074659

[72] Richardson M, Passmore H-A, Barbett L, Lumber R, Thomas R, Hunt A. The green care code: How nature connectedness and simple activities help explain pro-nature conservation behaviours. People Nat. 2020; 2(3): 821–39. 10.1002/pan3.10117

[73] Rudner J, Malone K. Childhood in the suburbs and the Australian Dream: How has it impacted children’s independent mobility? Glob Stud Child. 2011; 1(3): 207–25. doi: 10.2304/gsch.2011.1.3.207

[74] Davis JL, Green JD, Reed A. Interdependence with the environment: Commitment, interconnectedness, and environmental behavior. J Environ Psychol. 2009; 29(2): 173–80. doi: 10.1016/j.jenvp.2008.11.001

[75] Schultz PW. Inclusion with nature: The psychology of human-nature relations. In: Schmuck P, Schultz WP, editors. Psychology of sustainable development. Boston, MA: Springer; 2002. p. 61–78. doi: 10.1007/978-1-4615-0995-0_4

[76] Phenice LA, Griffore RJ. Young children and the natural world. Contemp Issues Early Child. 2003; 4(2): 167–71. doi: 10.2304/ciec.2003.4.2.6

[77] Carhart-Harris RL, Leech R, Hellyer PJ, Shanahan M, Feilding A, Tagliazucchi E, et al. The entropic brain: a theory of conscious states informed by neuroimaging research with psychedelic drugs. Front Hum Neurosci. 2014; 8(20): 1–22. doi: 10.3389/fnhum.2014.00020 24550805 PMC3909994

[78] Lyons T, Carhart-Harris RL. Increased nature relatedness and decreased authoritarian political views after psilocybin for treatment-resistant depression. J Psychopharmacol (Oxf). 2018; 32(7): 811–9. doi: 10.1177/0269881117748902 29338538 PMC6047302

[79] Peper JS, Burke SM, Wierenga LM. Sex differences and brain development during puberty and adolescence. In: Handbook of clinical neurology. Elsevier; 2020. p. 25–54. doi: 10.1016/B978-0-444-64123-6.00003–5 33008529

[80] Eames C, Barker M, Scarff C. Priorities, identity and the environment: Negotiating the early teenage years. J Environ Educ. 2018; 49(3): 189–206. doi: 10.1080/00958964.2017.1415195

[81] Liu T, Geng L, Ye L, Zhou K. “Mother Nature” enhances connectedness to nature and pro-environmental behavior. J Environ Psychol. 2019; 61: 37–45. doi: 10.1016/j.jenvp.2018.12.003

[82] Hunter LM, Hatch A, Johnson A. Cross-national gender variation in environmental behaviors. Soc Sci Q. 2004; 85(3): 677–94. doi: 10.1111/j.0038-4941.2004.00239.x

[83] Meinhold JL, Malkus AJ. Adolescent environmental behaviors: Can knowledge, attitudes, and self-efficacy make a difference? Environ Behav. 2016; 37(4): 511–32. doi: 10.1177/0013916504269665

[84] Nurse GA, Benfield J, Bell PA. Women engaging the natural world: Motivation for sensory pleasure may account for gender differences. Ecopsychology. 2010; 2(3): 171–8. doi: 10.1089/eco.2010.0025

[85] Eisenberger R, Sucharski IL, Yalowitz S, Kent RJ, Loomis RJ, Jones JR, et al. The motive for sensory pleasure: Enjoyment of nature and its representation in painting, music, and literature. J Pers. 2010; 78(2): 599–638. doi: 10.1111/j.1467-6494.2010.00628.x 20433632

[86] Lekies KS, Beery TH. Everyone needs a rock: Collecting items from nature in childhood. Child Youth Environ. 2013; 23(3): 66–88. doi: 10.7721/chilyoutenvi.23.3.0066

[87] Given L. The SAGE encyclopedia of qualitative research methods. Thousand Oaks, CA: SAGE Publications; 2008. doi: 10.4135/9781412963909

[88] Dickinson E. The misdiagnosis: Rethinking “nature-deficit disorder”. Environ Commun. 2013; 7(3): 315–35. doi: 10.1080/17524032.2013.802704

[89] Gray T. Outdoor learning: not new, just newly important. Curric Perspect. 2018; 38(2): 145–9. doi: 10.1007/s41297-018-0054-x

[90] Passy R, Bentsen P, Gray T, Ho S. Integrating outdoor learning into the curriculum: an exploration in four nations. Curric Perspect. 2019; 39(1): 73–8. doi: 10.1007/s41297-019-00070-8

[91] Beery T, Jørgensen KA. Children in nature: sensory engagement and the experience of biodiversity. Environ Educ Res. 2018; 24(1): 13–25. doi: 10.1080/13504622.2016.1250149

