# Supplementary figures and images for: Urban children’s connections to nature and environmental behaviors differ with age and gender
Source: PLoS One. 2021 Jul 29;16(7):e0255421. doi: 10.1371/journal.pone.0255421 (PMC8321113; doi:10.1371/journal.pone.0255421)

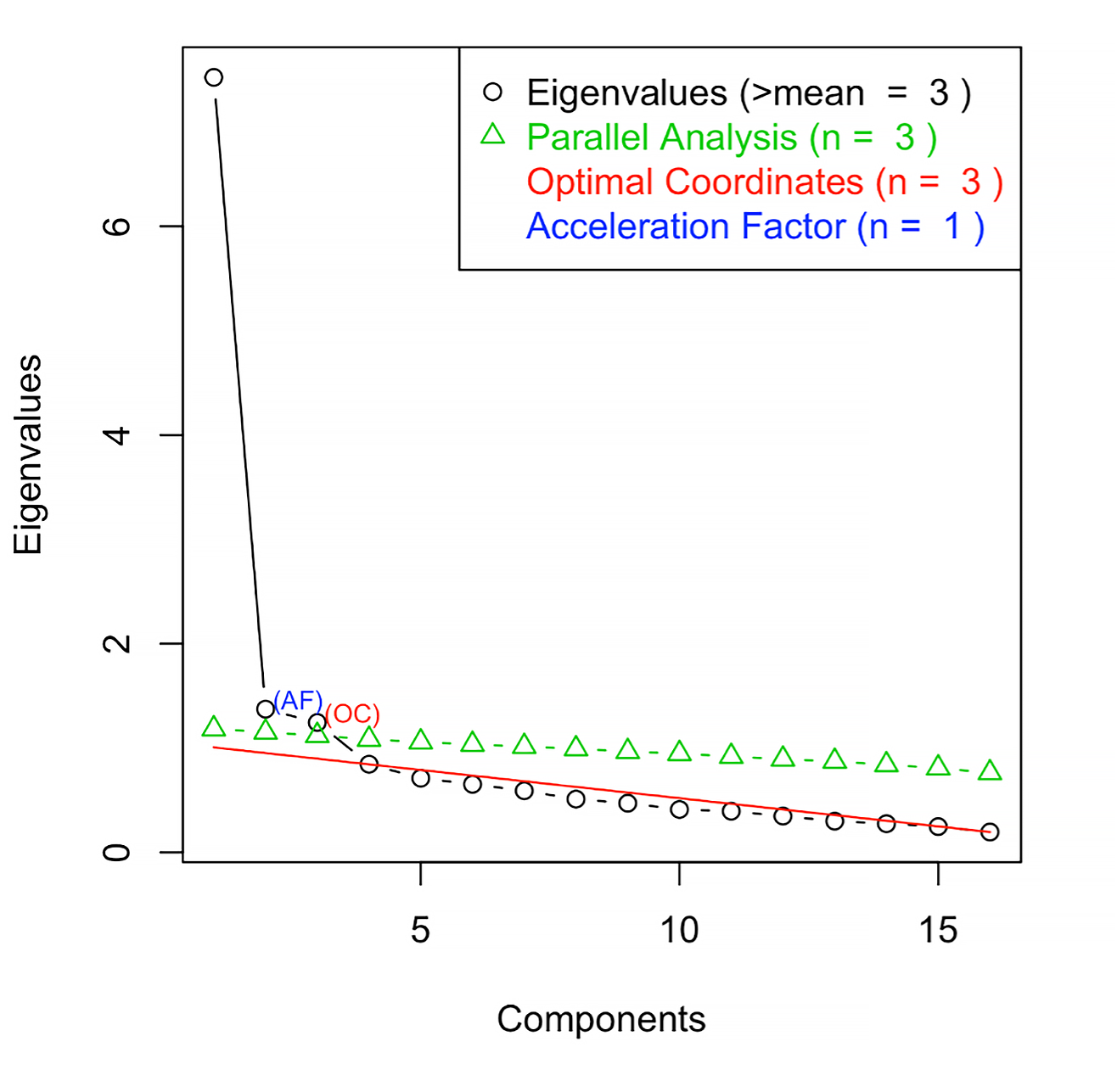

Supplement: S1 Fig — (TIF) [file pone.0255421.s001.tif]

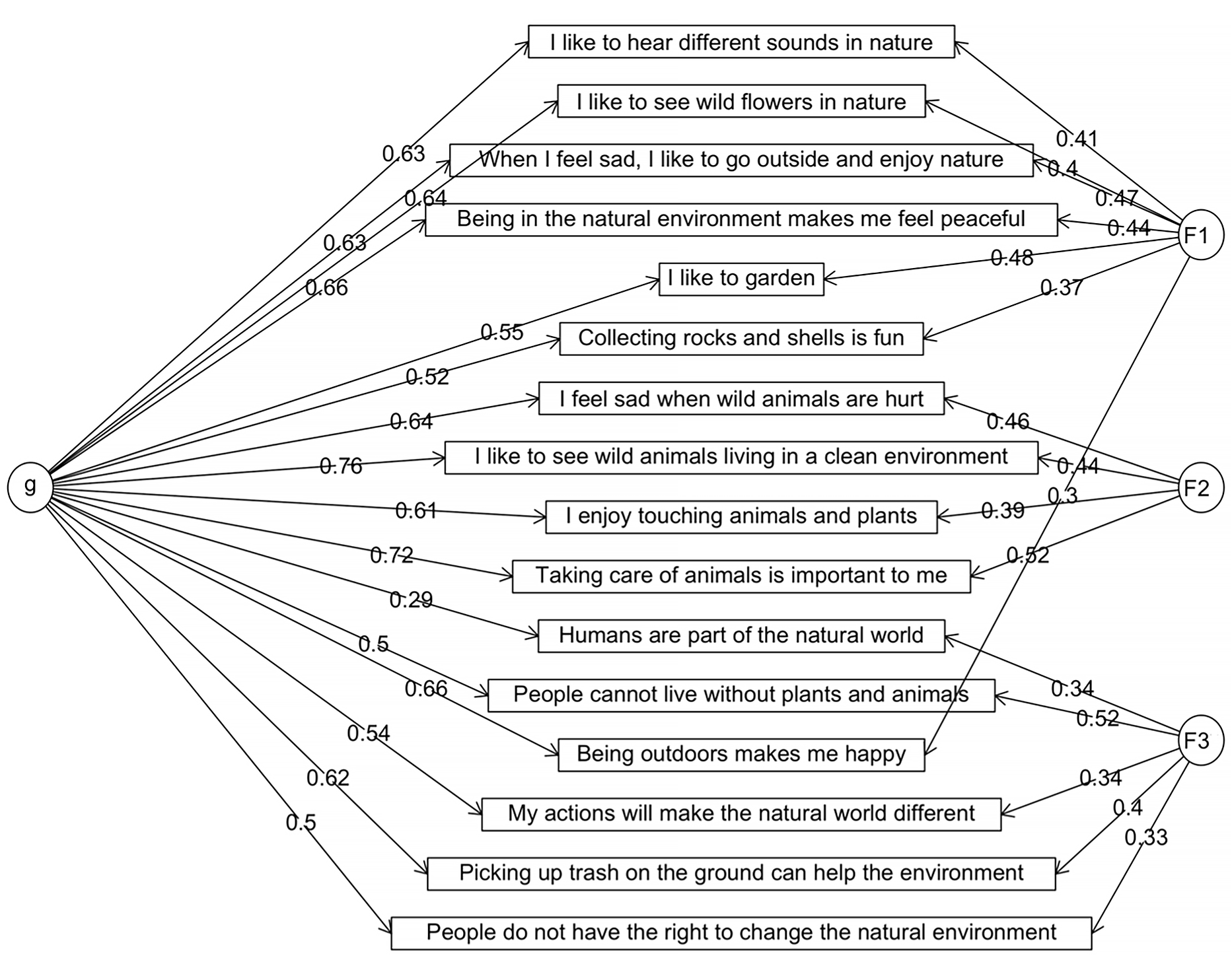

Supplement: S2 Fig — Paths are drawn where the absolute value of the Schmid-Leiman factor loading exceeds 0.2. (TIF) [file pone.0255421.s002.tif]

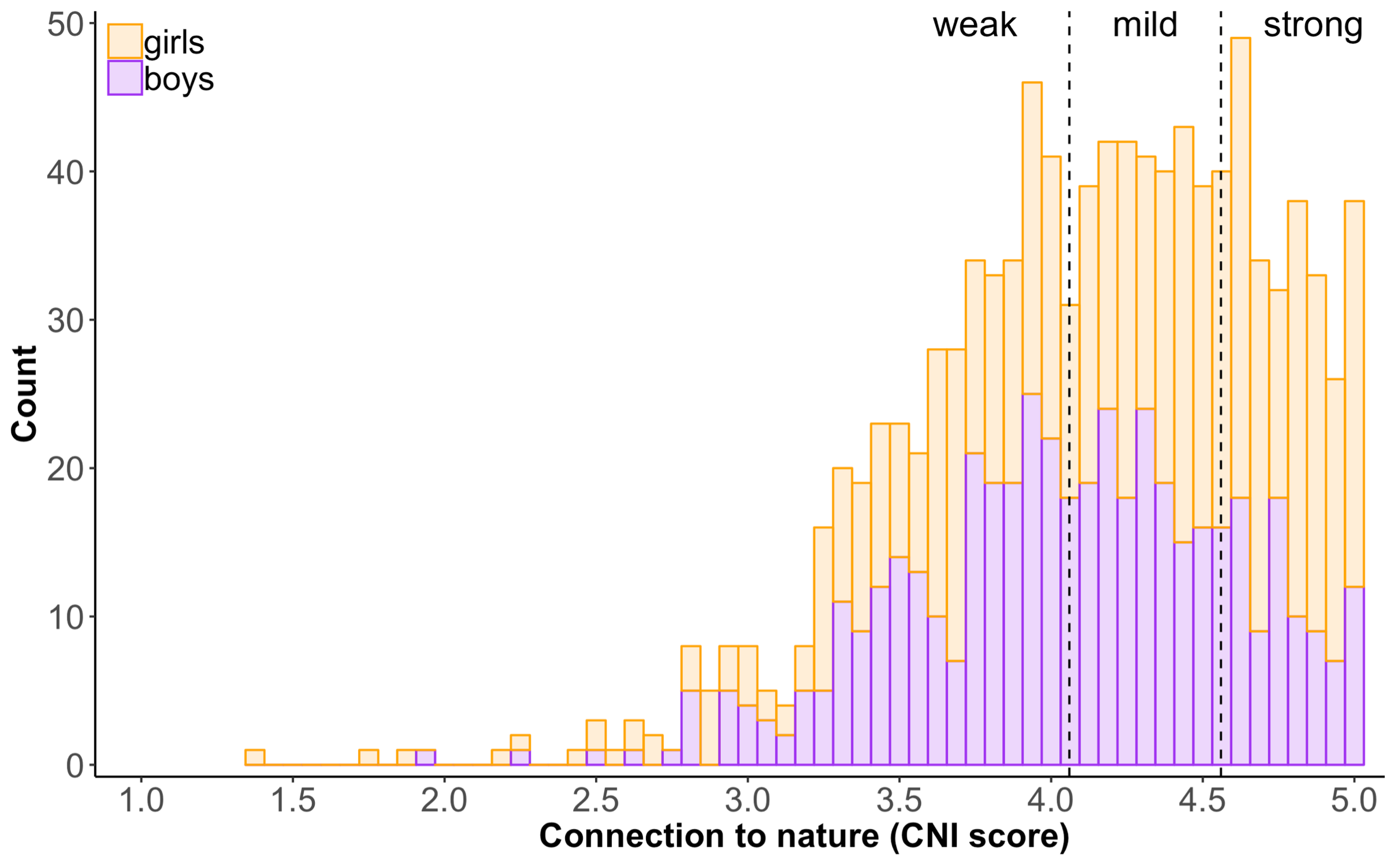

Supplement: S3 Fig — (TIF) [file pone.0255421.s003.tif]

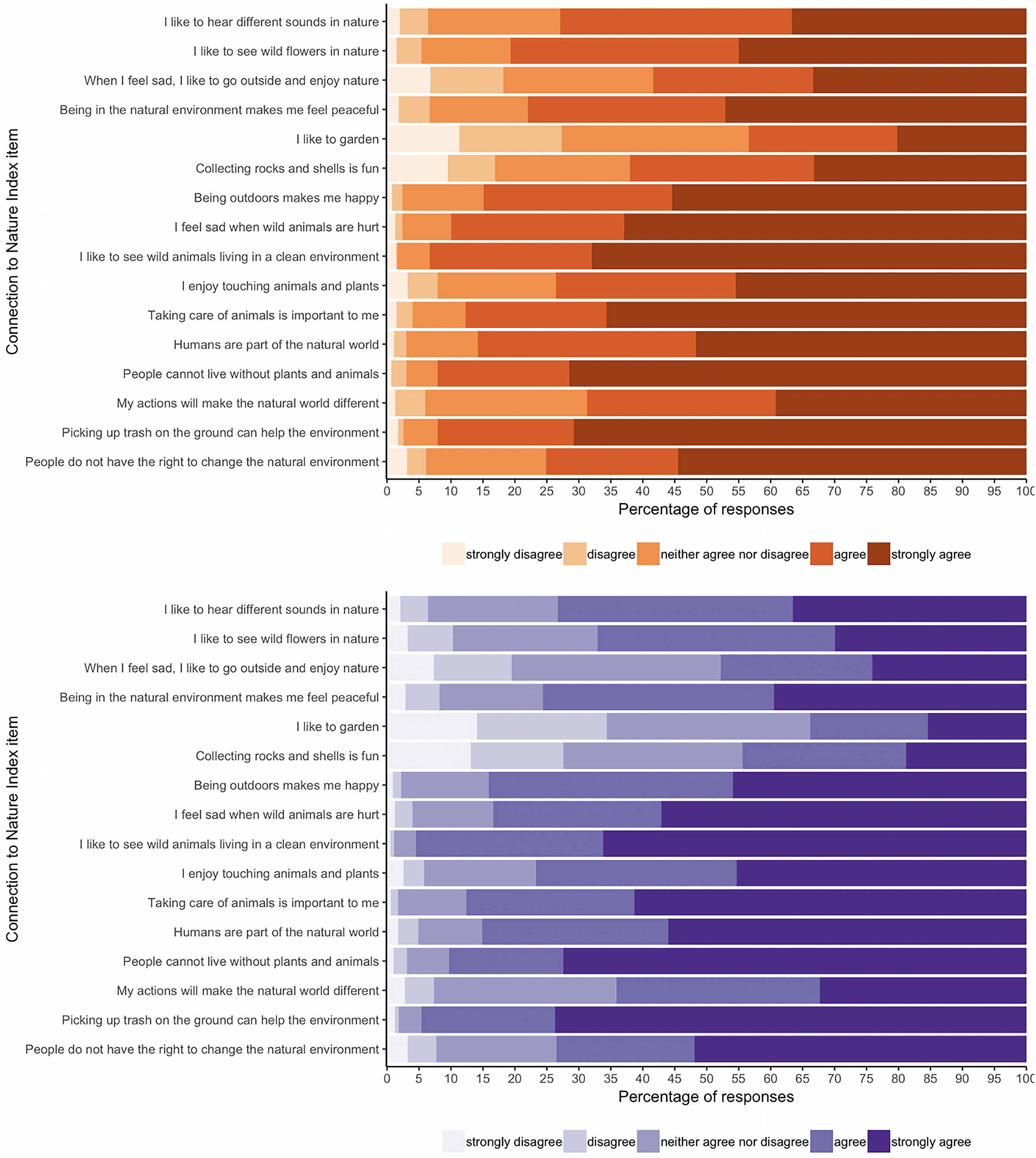

Supplement: S4 Fig — Items are ordered according to where they conventionally appear on the CNI. (TIF) [file pone.0255421.s004.tif]
